# Modulation of Amyloid β-Induced Microglia Activation and Neuronal Cell Death by Curcumin and Analogues

**DOI:** 10.3390/ijms23084381

**Published:** 2022-04-15

**Authors:** Ersilia De Lorenzi, Davide Franceschini, Cecilia Contardi, Rita Maria Concetta Di Martino, Francesca Seghetti, Massimo Serra, Federica Bisceglia, Andrea Pagetta, Morena Zusso, Federica Belluti

**Affiliations:** 1 Department of Drug Sciences, University of Pavia, 27100 Pavia, Italy; ersidelo@unipv.it (E.D.L.); cecilia.contardi01@universitadipavia.it (C.C.); massimo.serra@unipv.it (M.S.); federica.bisceglia@unipv.it (F.B.); 2 Department of Pharmaceutical and Pharmacological Sciences, University of Padua, 35131 Padua, Italy; dfrancesc@gmail.com (D.F.); andrea.pagetta@unipd.it (A.P.); 3 Department of Pharmacy and Biotechnology, Alma Mater Studiorum-University of Bologna, 40126 Bologna, Italy; rita.dimartino@uniupo.it (R.M.C.D.M.); francesca.seghetti2@unibo.it (F.S.); federica.belluti@unibo.it (F.B.)

**Keywords:** Alzheimer’s disease, amyloid β oligomers, microglia activation, neuronal toxicity, curcumin analogues

## Abstract

Alzheimer’s disease (AD) is a progressive neurodegenerative disorder that is not restricted to the neuronal compartment but includes important interactions with immune cells, including microglia. Protein aggregates, common pathological hallmarks of AD, bind to pattern recognition receptors on microglia and trigger an inflammatory response, which contributes to disease progression and severity. In this context, curcumin is emerging as a potential drug candidate able to affect multiple key pathways implicated in AD, including neuroinflammation. Therefore, we studied the effect of curcumin and its structurally related analogues **cur6** and **cur16** on amyloid-β (Aβ)-induced microglia activation and neuronal cell death, as well as their effect on the modulation of Aβ aggregation. Primary cortical microglia and neurons were exposed to two different populations of Aβ42 oligomers (Aβ42Os) where the oligomeric state had been assigned by capillary electrophoresis and ultrafiltration. When stimulated with high molecular weight Aβ42Os, microglia released proinflammatory cytokines that led to early neuronal cell death. The studied compounds exerted an anti-inflammatory effect on high molecular weight Aβ42O-stimulated microglia and possibly inhibited microglia-mediated neuronal cell toxicity. Furthermore, the tested compounds demonstrated antioligomeric activity during the process of in vitro Aβ42 aggregation. These findings could be investigated further and used for the optimization of multipotent candidate molecules for AD treatment.

## 1. Introduction

Alzheimer’s disease (AD) is the cause of 50–70% of dementias, and it is clinically characterized by cognitive impairment and memory loss [1]. The two major histopathological hallmarks of AD are intracellular neurofibrillary tangles and extracellular deposits of amyloid plaques [2]. Amyloid-β (Aβ) is a peptide generated by the sequential cleavage of amyloid precursor proteins by β- and γ-secretase enzymes. The two major isoforms of Aβ are Aβ40 (40-residue long) and Aβ42 (42-residue long), the latter bearing two extra residues at the C-terminus. Aβ42 is the major component of the amyloid plaques in AD, while Aβ40 is mainly present in the cerebrospinal fluid, suggesting that Aβ42 deposition precedes that of Aβ40 [3]. Aβ42 has a high rate of aggregation and self-assembly in progressively higher molecular weight structures (i.e., soluble oligomers and insoluble fibrils) [4,5]. It has become increasingly evident that Aβ monomers do not induce direct cellular toxicity at physiological concentrations, while soluble Aβ oligomers, rather than insoluble fibrils, are responsible for synaptotoxicity and neuronal cell damage. In fact, numerous in vitro and in vivo studies have shown that soluble Aβ oligomers induce various pathological alterations, including mitochondrial dysfunction, synaptic damage, oxidative stress, apoptosis, and cognitive deficits [5,6,7,8,9,10,11,12,13,14].

In addition to direct neurotoxic effects, Aβ also has the ability to bind to pattern recognition receptors on glial cells, including astrocytes and microglia, hence it contributes to the progression and severity of AD [15,16]. Microglia are the resident macrophages of the central nervous system (CNS) that rapidly activate in response to endogenous and exogenous stimuli, such as neuronal injury, invading pathogens, toxic compounds, or protein aggregates. Microglia participate in AD by clearing Aβ deposits and initiating phagocytosis to restore tissue homeostasis [17,18]. However, when the activation becomes chronic, microglia release cytotoxic mediators, including proinflammatory cytokines (e.g., interleukin (IL)-1β, IL-6, IL-8, tumor necrosis factor (TNF)-α), chemokines, complement components, and reactive oxygen and nitrogen species, resulting in neuronal cell degeneration [19,20]. Furthermore, serum and brain tissue of AD patients show increased levels of proinflammatory cytokines, such as IL-6 or TNF-α [21], suggesting that microglia activation and neuroinflammation play significant roles in AD. In this scenario, the modulation of microglia-mediated neuroinflammation along with the inhibition of Aβ oligomer formation by small molecules can represent a valuable therapeutic strategy for AD. In the past five decades, the design of multipotent compounds, able to concurrently modulate multiple critical AD pathological features, has become the guide of exhaustive medicinal chemistry efforts, thus experiencing an ascending evolution (for a pioneering concept of multi-target agents see [22,23]).

Curcumin, (diferuloylmethane), the primary active component isolated from the rhizome of *Curcuma longa*, exhibits multiple pharmacological effects, including the ability to suppress oxidative damage, neuroinflammation, and Aβ aggregation and deposition [24,25,26,27,28,29,30]. All of these biological activities, along with its neuroprotective effects from diversified mechanisms, contribute to the reported procognitive properties of curcumin [31]. However, chemical instability, poor water solubility, rapid metabolism, and rapid systemic elimination represent the main criticisms of curcumin’s clinical efficacy [32,33]. For this reason, intense efforts have been spent in the search for effective medicinal chemistry approaches to overcome the limitations of curcumin. In this scenario, a number of properly modified curcumin derivatives endowed with an improved biological profile have been developed, confirming the significant potential of the curcuminoid scaffold as previously documented [27,34]. Particularly evident outcomes have been obtained in the anticancer and anti-neurodegenerative therapeutic areas [30,35,36,37].

**Figure 1:**
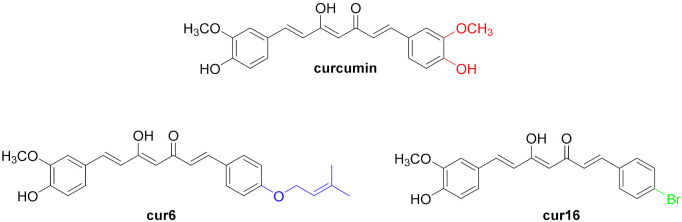
Chemical structures of curcumin and its analogues **cur6** and **cur16**.

In this study, by continuing our interest in developing curcumin-based analogues endowed with anti-neuroinflammatory properties and able to affect Aβ42 oligomer (Aβ42O) formation, we rationally designed **cur16**, a derivative structurally related to the previously reported **cur6** [30] (Figure 1).

In the effort to combine AD features, namely Aβ oligomer formation, with microglia activation and subsequent neuronal cell death, we employed an in vitro approach that used primary cortical microglia and neuronal cells exposed to Aβ42Os. We demonstrated that, when stimulated with high molecular weight (HMW) Aβ42Os, microglia released proinflammatory cytokines that seem to lead to early neuronal cell death. We employed this experimental setting to assess the effect of curcumin and the two structural analogues **cur6** and **cur16**, in terms of anti-inflammatory activity on HMW Aβ42O-stimulated microglia and microglia-mediated neuronal cell toxicity. Finally, we also evaluated the compounds for their capability to modulate during Aβ42 aggregation in vitro, the formation of toxic HMW Aβ42Os, and non-toxic low molecular weight (LMW) Aβ42Os.

## 2. Results

### 2.1. Rational Design and Synthesis of the Curcumin Analogues

Following a similar design rationale applied for **cur6**, we designed **cur16** in which both the central linker and the 4-hydroxy,3-methoxy aril ring were maintained as they proved to be optimal for the inhibition of Aβ aggregation and the release of microglial proinflammatory cytokines [30,38]. Whereas, the 3,3-dimethylallyloxy side chain was replaced with a bromine atom, regarded as a heavy halogen (with the equivalent weight of 6.7 carbon atoms) able to form and stabilize several interactions, among others halogen-bonding which is useful for improving the interaction of the new molecule with biological target [39]. Moreover, bromine, having steric, electric, and induction effects different from the 3,3-dimethylallyloxy function, affects lipophilic, electronic, and steric properties of **cur16**, thus modifying its spatial distribution and capability to interact with Aβ protein [40] (Supplementary Table S1).

**Scheme 1:**
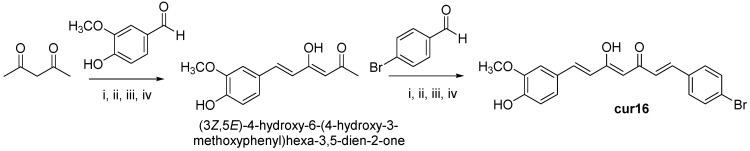
Synthetic route for **cur16**. Reagents and conditions: (**i**) B_2_O_3_, EtOAc; (**ii**) B(n-BuO)_3_; (**iii**) n-BuNH_2_, 80 °C; (**iv**) HCl, 80 °C.

**Cur6** was obtained as previously reported [30]. Similarly, **cur16** whose synthesis is reported in the PhD thesis of Dr. R.M.C. Di Martino, was prepared by performing a modified two-step Pabon reaction approach (Scheme 1) [35]. In detail, the semi-curcumin intermediate (3*Z*,5*E*)-4-hydroxy-6-(4-hydroxy-3-methoxyphenyl)hexa-3,5-dien-2-one, synthesized from the reaction between pentane-2,4-dione and 3-hydroxy,4-methoxy benzaldehyde, was reacted with 4-bromobenzaldehyde. The reaction course was monitored by thin-layer chromatography, and the obtained crude mixture was purified by flash column chromatography. The synthesized molecules were structurally characterized by ^1^H and ^13^C NMR spectroscopy and HRMS spectrometry. The final compounds, subjected to pharmacological evaluation, were characterized by a purity of ≥95% as evidenced by LC-MS analysis (Supplementary Figures S1 and S2).

### 2.2. Effect of Aβ42 Oligomers on Microglia Cell Viability

**Figure 2:**
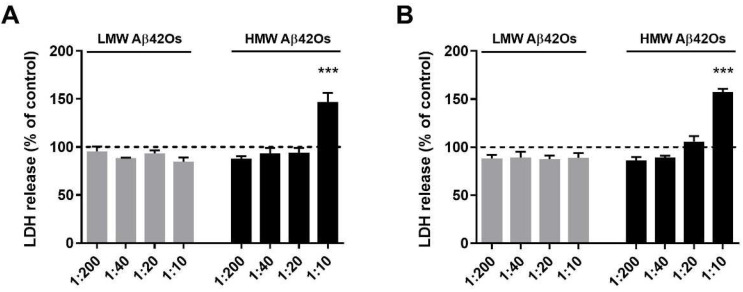
Effect of Aβ42 oligomers on microglia cell viability. Microglia were cultured overnight in medium containing 10% serum, which was replaced with serum-free medium before treatment with vehicle, LMW or HMW Aβ42Os for 24 (**A**) or 48 (**B**) h. At the end of incubation, cell viability was determined by LDH assay. Results are expressed as percentage of LDH release relative to control cells (dashed lines). Data are means ± SEM of 3 independent experiments, each performed in triplicate. *** *p* ˂ 0.001 versus control cells. Two-way ANOVA, followed by Tukey’s multiple comparison test.

To assess the effect of Aβ42Os on microglia viability, cells were treated with a serial dilution of LMW and HMW Aβ42Os (1:200, 1:40, 1:20, and 1:10) for 24 and 48 h. No cytotoxic effects were observed following treatment of microglia with all the tested dilutions of LMW Aβ42Os, as well as with 1:200, 1:40, and 1:20 dilutions of HMW Aβ42Os. However, the 1:10 dilution of HMW Aβ42Os resulted in a significant release of LDH after 24 and 48 h treatment, indicating a marked increase in cell toxicity (Figure 2). Based on these results, the dilution of 1:10 was excluded from further experiments.

### 2.3. Effect of Aβ42 Oligomers on Proinflammatory Cytokine Release from Microglia

**Figure 3:**
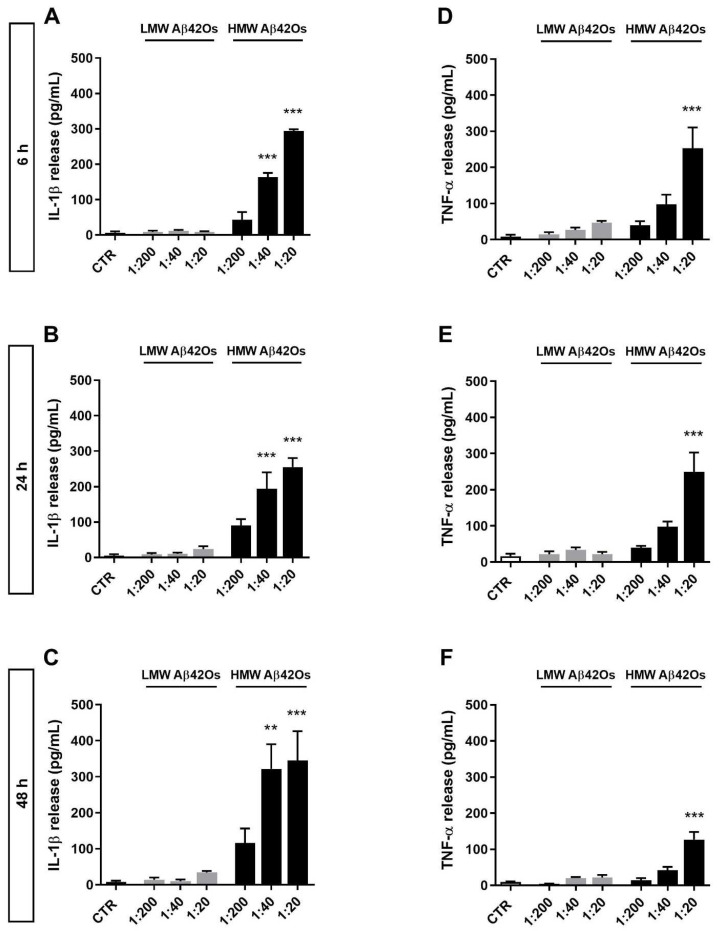
Effect of Aβ42 oligomers on cytokine release from cortical microglia. Microglia were cultured overnight in medium containing 10% serum, which was replaced with serum-free medium before treatment with vehicle, or a serial dilution of LMW and HMW Aβ42Os for 6, 24, or 48 h. Supernatants were collected and analyzed for IL-1β (**A**–**C**) and TNF-α (**D**–**F**) release by ELISA. Data are means ± SEM (n = 3 in triplicate). ** *p* ˂ 0.01, and *** *p* ˂ 0.001 versus control cells. Two-way ANOVA, followed by Tukey’s multiple comparison test.

Next, to explore the effect of LMW and HMW Aβ42Os on microglia inflammatory responses, the levels of two proinflammatory cytokines in culture supernatant were measured by ELISA. The exposure of HMW Aβ42Os for 6, 24, and 48 h enhanced the release of IL-1β and TNF-α in a concentration-dependent manner. Specifically, after a 6 h incubation with HMW Aβ42Os, a significant increase in IL-1β levels was observed starting from the dilution of 1:40. HMW Aβ42Os also increased the release of TNF-α at the dilution of 1:20 compared to control cells. In contrast, LMW Aβ42Os did not significantly modify basal levels of both cytokines (Figure 3).

### 2.4. Effect of Curcumin and Analogues on Aβ42 Oligomer-Induced Microglia Inflammatory Response

**Figure 4:**
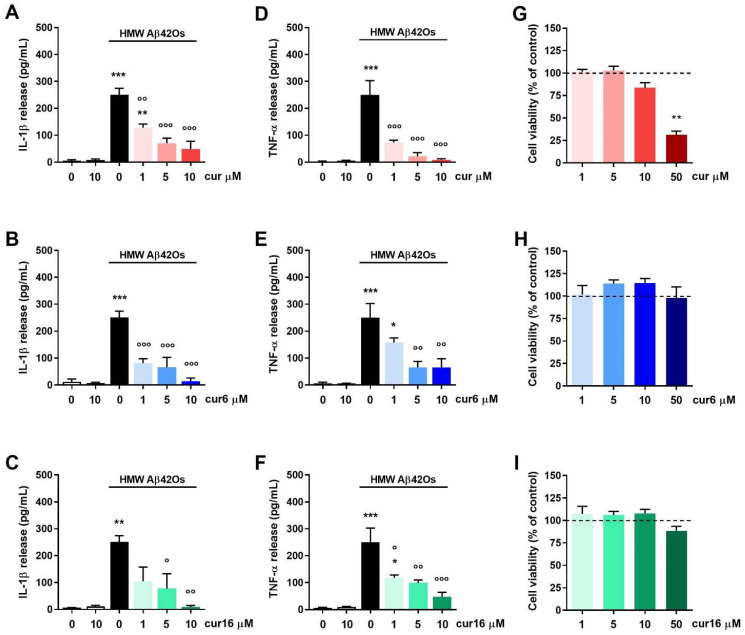
Effect of curcumin and its analogues on cytokine release from HMW Aβ42O-stimulated microglia and cell viability. Microglia were cultured overnight in medium containing 10% serum, which was replaced with serum-free medium before pretreatment with curcumin (cur), **cur6**, or **cur16** (1–10 μM) followed by stimulation with HMW Aβ42Os (1:20 dilution) for 24 h. Supernatants were collected and analyzed for IL-1β (**A**–**C**) and TNF-α (**D**–**F**) release by ELISA. (**G**–**I**) Cell viability was also determined by MTT assay. Results are expressed as percentage of cell viability relative to control cells (dashed lines). Data are means ± SEM (n = 3 in triplicate). * *p* ˂ 0.05, ** *p* ˂ 0.01, and *** *p* ˂ 0.001 versus control cells; ° *p* ˂ 0.05, °° *p* ˂ 0.01, and °°° *p* ˂ 0.001 versus HMW Aβ42O-treated cells. One-way ANOVA, followed by Holm–Sidak’s multiple comparison test.

We next examined whether curcumin and two structural analogues **cur6** and **cur16** could affect proinflammatory cytokine release induced by Aβ42O stimulation. For this analysis, cells were pretreated for 1 h with increasing concentrations (1–10 µM) of the three compounds before exposure to HMW Aβ42Os (1:20 dilution) for 24 h. Curcumin, **cur6**, and **cur16** alone at the highest non-cytotoxic concentration (i.e., 10 µM) did not modify the low amounts of IL-1β and TNF-α usually released by unstimulated microglia. Whereas, the three tested compounds inhibited the HMW Aβ42O-induced release of both the above cytokines in a concentration-dependent manner. The maximum inhibitory effect (i.e., an effect that did not differ significantly from the basal level) on IL-1β release was achieved with a 1 μM **cur6**, and a 5 μM curcumin and **cur16** treatment (Figure 4A–C). Analogously, TNF-α release was reduced to the control value by 1 μM curcumin and 5 μM **cur6** and **cur16** (Figure 4D–F). Taken together, these data show that curcumin, **cur6**, and **cur16** elicit an inhibitory effect on the secretion of the proinflammatory mediators IL-1β and TNF-α released by microglia after stimulation with HMW Aβ42Os.

Furthermore, it is worthy to note that the three compounds did not compromise cell viability at the concentrations tested (1–10 µM) (Figure 4G–I), thus indicating that the observed decrease in cytokine release did not result from cytotoxicity. In detail, the three compounds have been shown to affect cell viability differently with curcumin being the most toxic of the set. This behavior is generally ascribed to the reactive 4-hydroxy,3-methoxy aryl function that predominates in the symmetrical structure of curcumin, while **cur6** and **cur16** bear, on one aromatic ring, substituents with different steric and electronic properties.

### 2.5. Effect of Aβ42 Oligomers on Neuronal Cell Toxicity

**Figure 5:**
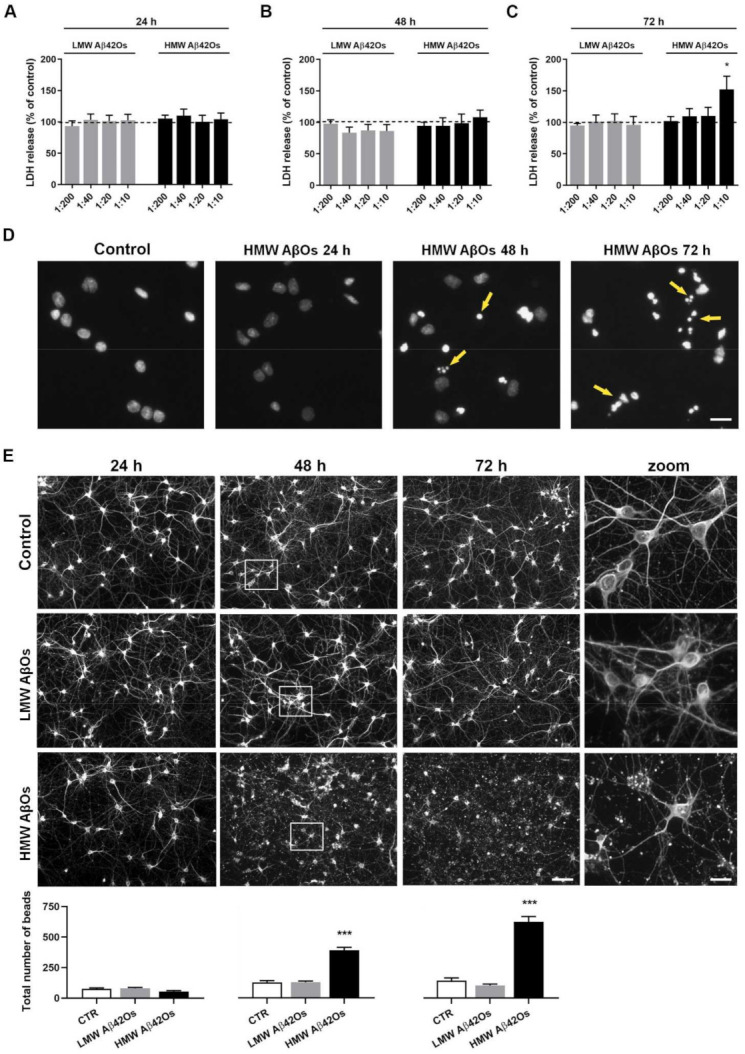
Effect of Aβ42 oligomers on neuronal cell toxicity. Primary cortical neurons were cultured from E17 rat embryos and after 7 days in vitro were exposed to a serial dilution of LMW or HMW Aβ42Os (1:200, 1:40, 1:20, and 1:10) for 24 (**A**), 48 (**B**), or 72 (**C**) h. At the end of incubation, cell viability was determined by LDH assay. Results are expressed as percentage of LDH release relative to control cells (dashed lines). Data are means ± SEM of 3 independent experiments, each performed in triplicate. * *p* ˂ 0.05 versus control cells. Two-way ANOVA, followed by Tukey’s multiple comparison test. (**D**) Primary neurons were treated with HMW Aβ42Os (1:10 dilution) for 24, 48, or 72 h. Cells were then processed for DAPI staining. Yellow arrows indicate cells with small, fragmented, and condensed nuclei. Experiments were performed 3 times and representative fluorescence microscopy images are shown. The scale bar is 10 µm. (**E**) Primary neurons were treated with LMW or HMW Aβ42Os (1:10 dilution) for 24, 48, or 72 h. Cells were then processed for βIII-tubulin immunostaining. Experiments were performed 3 times and representative fluorescence microscopy images are shown. The scale bar on the left is 50 µm. In the zoomed images, the scale bar is 10 µm. Quantification of neuritic beading, performed using ImageJ software, is shown at the bottom of the corresponding images. Data are means ± SEM from five random fields of 3 independent experiments. *** *p* ˂ 0.001 versus control cells. One-way ANOVA, followed by Holm–Sidak’s multiple comparison test.

We then tested the toxicity of Aβ42Os on primary cortical neurons. Initially, to analyze the direct effect of Aβ42Os, neurons were treated with different dilutions of LMW and HMW Aβ42Os (1:200, 1:40, 1:20, and 1:10) for 24, 48, or 72 h. The quantification of LDH released into the culture media showed that only HMW Aβ42Os at the highest concentration tested (i.e., 1:10 dilution) induced significant neuronal toxicity after 72 h treatment (Figure 5A–C). However, since LDH assay reflects the disruption of the plasma membrane that happens during necrosis and late apoptosis [41], we also verified the presence of alterations in the nuclear morphology, as indicators of activation of programmed cell death. To test this, neurons treated with 1:10 dilution of HMW Aβ42Os were stained with 4′,6-diamidino-2-phenylindole (DAPI), which has often been used to reveal chromatin condensation and nuclear breakdown as features of apoptotic cell death [42]. Figure 5D shows that nuclei of control and HMW Aβ42O-treated cells for 24 h appeared uniform in size and homogeneously stained with moderate intensity. Whereas treatment with HMW Aβ42Os for 48 and 72 h showed an increased number of cells with small, fragmented, and condensed nuclei (see yellow arrows), indicating an increase of apoptotic cells.

Considering that previous studies have shown that Aβ oligomers cause pathological changes in neuronal morphology that include microtubule disruption and neuritic beading [43,44], cells were stained with βIII-tubulin to analyze their morphology after treatment with Aβ42Os (1:10 dilution) for 24, 48, and 72 h. As shown in Figure 5E, control cells, neurons treated with LMW Aβ42Os for 24, 48, and 72 h as well as with HMW Aβ42Os for 24 h, exhibited intact cell bodies with elaborate networks of neurites. On the contrary, neurons treated with HMW Aβ42Os for 48 h displayed processes with numerous βIII-tubulin accumulations (Figure 5E), typical of neuritic beading that reflect neuritic damage. After 72 h of HMW Aβ42O treatment, the neuronal network appeared to be collapsed (Figure 5E).

**Figure 6:**
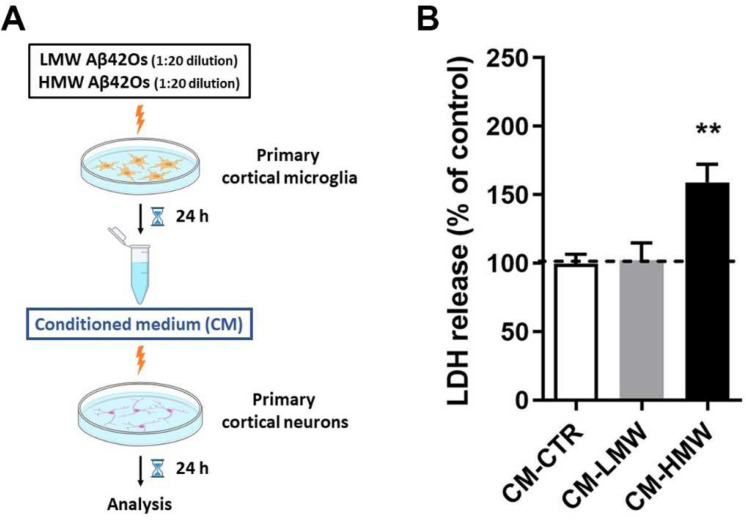
Effect of microglia-conditioned media on neuronal cell toxicity. (**A**) The procedure of collecting microglia-conditioned media to stimulate primary cortical neurons. (**B**) Primary cortical neurons were cultured from E17 rat embryos and after 7 days in vitro were exposed for 24 h to conditioned media from microglia untreated (CM-CTR) or treated with 1:20 dilution of LMW (CM-LMW) or HMW (CM-HMW) Aβ42Os for 24 h. At the end of incubation, cell toxicity was determined by LDH assay. Results are expressed as percentage of LDH release relative to control cells (dashed line). Data are means ± SEM of 3 independent experiments, each performed in duplicate. ** *p* ˂ 0.01 versus control cells. One-way ANOVA, followed by Holm–Sidak’s multiple comparison test.

Previous studies have shown that, in addition to direct neuronal cell death, Aβ can cause indirect neurotoxicity mediated by activated microglia that secrete toxic factors, including reactive oxygen species, nitric oxide, and cytokines [45,46,47]. To test this, primary neurons were treated for 24 h with conditioned media obtained from microglia exposed to 1:20 dilution of Aβ42Os for 24 h (Figure 6A). No cytotoxic effects were observed following the treatment of neurons with conditioned media obtained from microglia exposed to LMW Aβ42Os. Conversely, conditioned media from HMW Aβ42O-stimulated microglia increased neuritic damage (Supplementary Figure S3) and neuronal cell death (Figure 6B). Although a 24 h treatment with HMW Aβ42Os alone was not toxic to neurons (Figure 5A,D,E), it stimulated neuronal cell death in the presence of microglia-conditioned media, suggesting that inflammatory mediators secreted by Aβ42O-stimulated microglia can play a role in the early neuronal cell toxicity.

### 2.6. Effect of Curcumin and Analogues on Indirect Neuronal Cell Toxicity Induced by Aβ42 Oligomers

**Figure 7:**
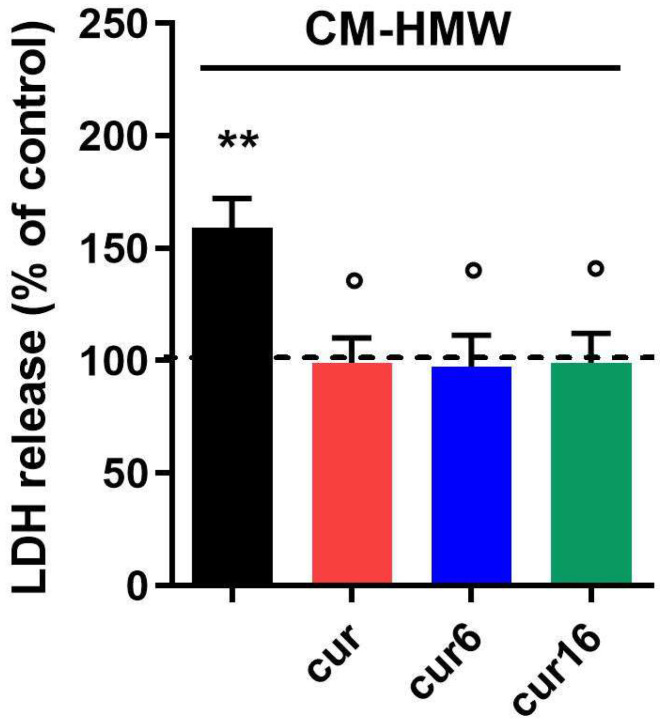
Effect of conditioned medium from microglia treated with curcumin and analogues on neuronal cell toxicity. Primary cortical neurons were cultured from E17 rat embryos and after 7 days in vitro were exposed for 24 h to conditioned media from microglia pretreated with 10 μM curcumin (cur), **cur6**, or **cur16** before stimulation with HMW (CM-HMW; 1:20 dilution) Aβ42Os for 24 h. At the end of incubation, cell viability was determined by LDH assay. Results are expressed as percentage of LDH release relative to control cells (dashed line). Data are means ± SEM of 3 independent experiments, each performed in duplicate. ** *p* ˂ 0.01 versus control cells and ° *p* ˂ 0.05 versus CM-HMW. One-way ANOVA, followed by Holm–Sidak’s multiple comparison test.

Since we found that curcumin, **cur6**, and **cur16** markedly reduced the release of inflammatory cytokines induced by HMW Aβ42Os, we next asked whether the three compounds could protect neurons from Aβ42 oligomers toxicity induced by microglia. For this purpose, conditioned media from microglia pretreated for 1 h with 10 µM curcumin, **cur6**, or **cur16** before stimulation with HMW Aβ42Os were added to primary cortical neurons for 24 h. Neurite damage and neuronal cell death were abolished when neurons were exposed to conditioned media from microglia pretreated with curcumin, **cur6**, or **cur16** (Figure 7 and Supplementary Figure S3), suggesting that neuronal cell death may be suppressed through the reduction of neuroinflammation induced by Aβ42Os.

### 2.7. Effect of Curcumin and Analogues on Aβ42 Oligomerization

**Figure 8:**
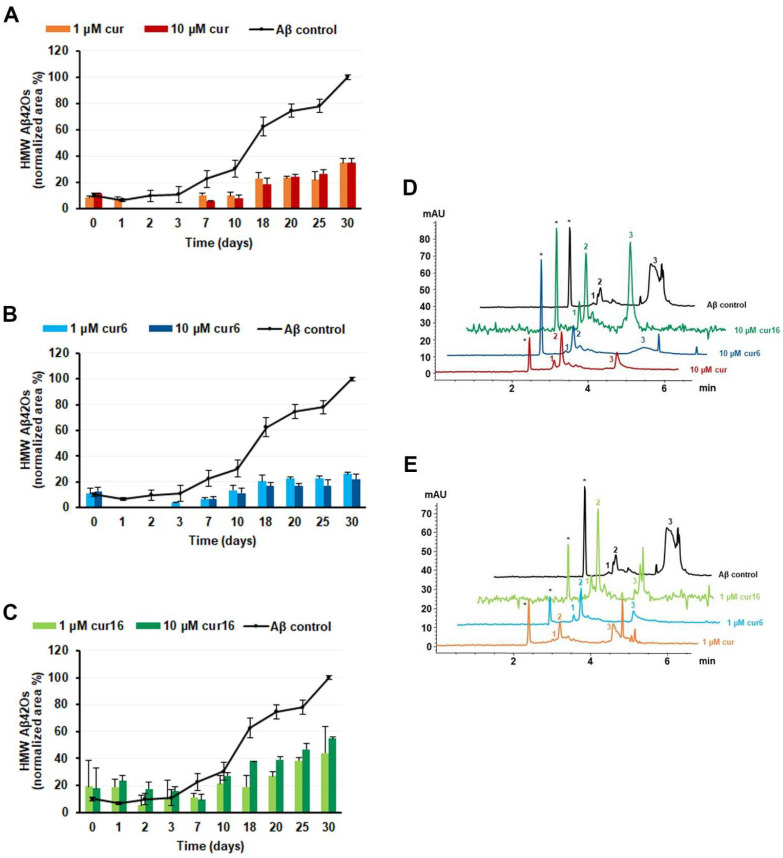
Effect of curcumin and analogues on Aβ42 oligomerization. (**A**–**C**) Normalized area percentage plots of toxic HMW Aβ42Os from a 221 μM solution of Aβ42 monomer, in the absence (control) and in the presence of decreasing concentrations of curcumin (cur, **A**), **cur6** (**B**) and **cur16** (**C**). Data are expressed as mean ± standard deviation (n = 3). (**D**,**E**) Comparison among electrophoretic profiles of 221 μM Aβ42 control (black traces) and in the presence of 10 μM (**D**) and 1 μM (**E**) curcumin, **cur6**, and **cur16**. Peak annotation: * electroosmotic flow; 1 and 2: LMW Aβ42Os; 3 HMW Aβ42Os. Electropherograms are taken at 25 days from dissolution and are representative of n = 3.

For this final evaluation, by using a sample preparation protocol previously established by us [48], the formation over time of Aβ42Os was monitored daily by capillary electrophoresis (CE), in the absence or presence of curcumin, **cur6**, and **cur16** analogues. The sample preparation was such as to induce the dynamic formation of the oligomers, before precipitation into fibrils, over a wide time window (30 days, Figure 8A–C). Notably, from the electrophoretic traces it was possible to appreciate if a compound either reduced the formation of the toxic HMW Aβ42Os, migrating under peak 3 or kept in solution for a longer time the non-toxic LMW Aβ42Os, migrating under faster peaks 1 and 2 [30]. In Figure 8D,E, peaks 1 and 2 correspond to Aβ monomers and dimers whereas peak 3 corresponds to soluble aggregates bigger than dodecamers; the toxicity on IMR-32 and SH-5YSY neuroblastoma cells of the latter species was well established in our previous studies [48,49].

Overall, Figure 8 shows that all tested compounds reduced the formation of toxic oligomers to a great extent when compared to the control, with **cur6** being the most active, even at a concentration as low as 1 μM.

## 3. Discussion

Soluble aggregates of Aβ peptide play a crucial role in AD and may represent the early and central event in the pathogenesis of the disease [14]. However, the structure of Aβ oligomers, the molecular mechanisms involved in the aggregation process, the mechanism of toxicity, and the identification of the neurotoxic oligomeric species are still largely unclear. Since Aβ oligomers are heterogeneous populations of polymorphic and non-covalent aggregates, their study is complicated and often leads to controversial results [12,50]. Over the years, Aβ aggregates and their toxicity have been studied using multiple techniques (e.g., SDS-PAGE, mass spectrometry, NMR spectroscopy, size exclusion chromatography, electron, and atomic force spectroscopy [51], dynamic and multiangle light scattering [52,53]) that can be subject to limitations or disadvantages. The use of CE for the detection and separation of soluble oligomeric species of Aβ peptides has been introduced by us [49]. In contrast to liquid chromatography or SDS-PAGE, CE works in the absence of a stationary phase or chaotropic agents. This affords a real-time snapshot of Aβ while assembling into soluble oligomers inside the capillary, until sample precipitation. Due to the unique possibility of preserving the native oligomeric structure in free solution, the advantage of this tool is three-fold: first, as widely reported by us and others, it finds application in assessing the potential antioligomeric activity of coincubated small molecules [30,54,55,56,57,58,59]; second, by ultrafiltration followed by CE analysis, it is possible to assign a molecular weight range to the species related to the electrophoretic peaks; third, the filtered and retained oligomers can be independently characterized by their structure and toxicity [48] or exploited for cell treatments. In this study, oligomers were prepared following a well-established protocol, described, and validated in [48]. To this end, the CE analysis served as an actual control for the reproducibility of the preparation, thus we can reasonably state that the filtrated portion (first electrophoretic peak) corresponds to aggregates from trimers up to dodecamers (LMW Aβ42Os) and the retained portion (second electrophoretic peak) to aggregates smaller than 22-mers and larger than dodecamers (HMW Aβ42Os) (Supplementary Figure S4). Consistently with their size, these two populations have shown a progressive increase in the β-sheet structure for HMW Aβ42Os, when compared to the higher content of α-helical/random coil structure of LMW Aβ42Os [48]. It has been recently reported [60] that the biological characteristics of Aβ42Os in the brain of AD patients are scarcely studied, due to the reasons mentioned above. Over the past twenty years, a wealth of in vitro and in vivo experiments have been carried out using Aβ42Os, also prepared from synthetic Aβ; these so-called chemical models of AD shed light on their multiple neurotoxic mechanisms. The latest in vivo studies showed that human brain extracts contain highly heterogeneous Aβ monomers and detergent-stable dimers, trimers, and higher oligomers [61]. Toxicity attribution to a specific species is still difficult, controversial, and confusing. For example, the lack of smaller species and the existence of toxic dodecamers and larger species were recently reported in the brain [62]; conversely, another study supported that, under certain conditions, the predominant HMW AβOs in the AD brain can dissociate into LMW species which are significantly more bioactive than the HMW species from which they are derived [63].

Here, we first analyzed the effect of LMW and HMW Aβ42Os in the inflammatory response of microglia, the primary players in the CNS immune response. Although extensive evidence shows that Aβ aggregates stimulate microglia activation, it is not sufficiently clear which form (s) of Aβ activates microglia. The great majority of studies have focused on the differences between Aβ oligomers and Aβ fibrils. For example, Sondag et al. [64] have shown that these two species stimulated similar but diverse signaling responses in microglia; furthermore, microglia secretory phenotypes stimulated by Aβ oligomers or Aβ fibrils were distinct. Unlike previous studies, here we used two specific populations of Aβ oligomers, where the oligomeric state had been assigned by CE and ultrafiltration. We demonstrated that only HMW Aβ42Os significantly increased microglia activation as revealed by the increased release of the proinflammatory cytokines IL-1β and TNF-α, suggesting that the supramolecular properties, the conformation, and presumably also the surface area of Aβ oligomers influence their ability to activate microglia.

Reactive microglia and increased levels of inflammatory mediators have been found to be closely associated with amyloid plaques in the brain of AD patients, indicating that an interaction exists between AD, microglia, and neuroinflammation [15,65]. Consequently, anti-inflammatory drugs have been proposed to suppress neuroinflammation in AD. However, several clinical trials showed the inefficacy of these drugs in symptomatic individuals with AD [66]. Considerable efforts have also been made with some natural products that have been used to delay the progression of the disease. In particular, several studies suggested that curcumin has antiamyloid as well as antioxidant and anti-inflammatory properties [24,25,28,29,30,67,68]. Several analogues of curcumin have also been synthesized and tested for their beneficial effects in AD models [69]. In our previous studies we have shown the inhibitory effect of curcumin and some of its analogues in LPS-induced microglia inflammatory response [25,28,29,30]. Here, we demonstrated that curcumin and the analogues **cur6** and **cur16** also inhibited the secretion of IL-1β and TNF-α by HMW Aβ42O-stimulated microglia.

Microglia and neurons participate in a bidirectional communication that is essential for brain homeostasis. In fact, dysregulated communication may result in CNS perturbation and neurodegenerative diseases. Under pathological conditions, such as AD, microglia produce neurotoxic molecules that can influence neuronal cell integrity, function, and survival [70]. Based on these observations, we investigated the impact of microglia on Aβ42O-mediated neuronal cell toxicity. To verify this, we analyzed both the direct toxicity and microglia-mediated toxicity of Aβ42Os on primary cortical neuronal cells. Treatment for 48 h with HMW Aβ42Os showed considerable beading and fragmentation of neurites associated with the presence of apoptotic cells. Interestingly, these effects were evident before the observation of a significant LDH release. The presence of an abnormal βIII-tubulin accumulation suggests that HMW Aβ42Os disrupt microtubule networks, indicative of dysfunctional axonal transport mechanisms. Neuritic beading has been seen in various pathological conditions, including patients with AD and transgenic mouse models of the disease, and has been considered a hallmark of neuronal cell toxicity [71]. When neurons were exposed to conditioned media from HMW Aβ42O-stimulated microglia, neuritic beading and cell death occurred prematurely (i.e., 24 h after exposure to conditioned medium), suggesting that inflammatory mediators secreted by Aβ42O-stimulated microglia can be responsible for early neuronal toxicity. In contrast, conditioned media from microglia exposed to HMW Aβ42Os in the presence of curcumin and the two analogues, protected neurons from toxic effect indicating that the suppression of Aβ42O neurotoxicity could result from the inhibition of microglia activation.

Finally, CE was also exploited to appreciate, over time, the effect of co-incubated curcumin, **cur6**, and **cur16** on the formation of Aβ42Os. In contrast to many tests carried out in search of modulators of Aβ42 oligomerization, this type of analysis in free solution preserves the transient nature, as well as the dynamic equilibrium, of the forming soluble aggregates. The peptide was injected at different elapsed times from solubilization and the progressive conversion over time of monomers and dimers into toxic, larger aggregates was monitored. As mentioned, all tested compounds reduced the toxic oligomers to a great extent, compared to the control. Notably, only **cur16** shifted the equilibrium towards the nontoxic monomers and dimers (LMW Aβ42Os) that were kept in solution at a higher concentration for a longer time. In this respect, **cur16** performed better than curcumin, whose capability to stabilize Aβ42 dimers was already reported [72]. It is also evident that **cur16** was the only compound whose antioligomeric activity was higher at 1 μM rather than 10 μM. The well-known ability of the curcumin scaffold to interact not only with the biological counterpart but also with itself leads to the formation of heteromolecular complexes, with modified activity. This behavior has been reported to be dependent on ligand concentration [73] and could elucidate the in vitro **cur16** bioactivity profile. Indeed, the bromine substituent can likely affect its self-assembling properties and, as a consequence, the interaction with Aβ42Os. Thus, at moderate-high **cur16** in vitro concentration and some buffers, an increased self-aggregating propensity of the molecule with a resultant deviation from the linear dose–response is speculated.

In conclusion, in this study we demonstrated the anti-inflammatory and antioligomeric properties of curcumin and the newly studied analogues **cur6** and **cur16**. Their ability to suppress HMW Aβ42O-induced microglia activation and inflammatory response also seems to exert protective effects on neurons in vitro. However, to corroborate our results, we should test whether the depletion of either Aβ or cytokines in the microglia-conditioned media affects neuronal cell toxicity, as already shown by Floden et al. [46]. Furthermore, despite that the exact mechanism underlying curcumin, **cur6**, and **cur16** neuroprotective activity needs to be determined, a direct effect on the microglia inflammatory response cannot be ruled out. It is anyway demonstrated that curcumin, **cur 6**, and **cur16**, albeit to a different extent, reduce the building-up of those very same oligomers (HMW Aβ42O) that we have demonstrated play a role in inflammation.

Overall, the results obtained with curcumin, **cur6**, and **cur16** help to gain insight into the rational design and optimization of multipotent anti-AD molecules.

## 4. Materials and Methods

### 4.1. Reagents

Aβ42 was purchased as a lyophilized powder from AnaSpec (Fremont, CA, USA) and stored at −21 °C. Ethanol 96° was provided by Carlo Erba (Cornaredo, Italy). NaOH and acetonitrile (ACN) were from AppliChem PanReac, ITW companies (Milan, Italy). Solutions were prepared using Millipore Direct-Q deionized water (Bedford, MA, USA) and filtered on 0.45 μm ABLUO^®^ Syringe Filters from GVS North America (Sandford, ME, USA). Uncoated fused silica capillaries were from Polymicro Technologies (Phoenix, AZ, USA). Curcumin (analytical standard, purity ≥98%) was purchased from Sigma-Aldrich (Milan, Italy). All samples containing curcumin and curcumin derivatives were kept protected from light throughout all experiments. Tissue culture media, B27 supplement, antibiotics, and fetal bovine serum (FBS) were obtained from Thermo Fisher Scientific (Waltham, MA, USA). Mouse anti-βIII tubulin primary antibody (Cat. MA1-118) and Alexa Fluor 488 secondary antibody (Cat. A32723) were from Invitrogen (Milan, Italy). Enzyme-linked immunosorbent assay (ELISA) kits were obtained from Antigenix America (Huntington Station, NY, USA). Falcon tissue culture plasticware were purchased from BD Biosciences (SACCO SRL, Cadorago (CO), Italy). All other reagents were from Sigma-Aldrich.

### 4.2. General Chemistry Procedures for **cur6** and **cur16** Synthesis

All reagents and solvents were purchased from commercial suppliers and were of high purity and analytical grades. Reaction crudes were analyzed by thin-layer chromatography (TLC) on precoated aluminum-backed silica gel sheets (Merck Silica Gel 60 F254, Merck, Darmastadt, Germany) followed by UV lamp visualization of the spots and purified by flash column chromatography on silica gel (Kieselgel 40, 0.040–0.063 mm, Merck). Recorded melting points were determined in open glass capillary tubes by a Büchi melting point apparatus (Büchi, Cornaredo, Italy). The ^1^H-NMR and ^13^C-NMR spectra were recorded with a 400 MHz Varian INOVA spectrometer (Varian, Palo Alto, CA, USA), reporting chemical shifts as δ values (parts per million, ppm). Spin multiplicity was reported following the standard abbreviations: s (singlet), d (doublet), dd (doublet of doublet), and br (broad). Coupling constants (*J*) were reported in Hertz (Hz). Mass spectra (MS) were recorded on a Waters ZQ 4000, XevoG2-XSQTof, Acquity arc-QDA LC-MS apparatus equipped with electrospray ionization (ESI) in positive mode (Waters, Milford, MA, USA). **Cur6** and **cur16** purity were determined by ultra-performance liquid chromatography (UPLC) on Waters ACQUITY ARCUHPLC/MS system consisting of a QDa mass spectrometer equipped with an electrospray ionization interface and a 2489 UV/Vis detector, using the area percentage method. The detected wavelengths (λ) were 254 nm and 365 nm. The analyses were performed on a Waters XBridge BEH C18 column (10 × 2.1 mm i.d., particle size 2.5 μm) with an XBridge BEH C18 VanGuard Cartridge precolumn (5 mm × 2.1 mm i.d., particle size 1.8 μm). The mobile phases were H_2_O (0.1% formic acid) (A) and MeCN (0.1% formic acid) (B). Electrospray ionization in positive and negative modes was applied in the mass scan range 50–1200 Da. The following method and gradients were used: Generic method. Linear gradient: 0–0.78 min, 20% B; 0.78–2.87 min, 20−95% B; 2.87–3.54 min, 95% B; 3.54–3.65 min, 95–20% B; 3.65-5-73, 20% B. Flow rate: 0.8 mL/min. Compounds were named using the PerkinElmer Chem Office Suite 2020 IUPAC name algorithm (PerkinElmer Inc., Waltham, MA, USA).

### 4.3. Synthesis of **cur16** ((1E,4Z,6E)-1-(4-bromophenyl)-5-hydroxy-7-(4-hydroxy-3-methoxyphenyl)hepta-1,4,6-trien-3-one)

To a stirred solution of 3*Z*,5*E*-4-hydroxy-6-(4-hydroxy-3-methoxyphenyl)hexa-3,5-dien-2-one (0.43 g, 1.84 mmol) in ethyl acetate (EtOAc, 1.9 mL), boron oxide (B_2_O_3_, 0.13g, 1.84 mmol) was added, and the suspension was stirred for 30 min at 80 °C. Then 4-bromobenzaldehyde (0.30 g, 1.66 mmol) was added, followed by tri-n-butyl borate ([CH_3_(CH_2_)_3_O]_3_B, 0.85 g, 0.61 mL, 3.68 mmol) in EtOAc (0.8 mL). The reaction mixture was heated at 80 °C for 30 min, then a solution of 1-butylamine (n-BuNH_2_, 0.27 g, 0.37 mL, 3.68 mmol) in EtOAc (2.0 mL) was added over a 15 min period, and the heating was continued for 4 h. After cooling to room temperature, 0.5 N HCl (30 mL) was added, and then the reaction was stirred at room temperature for 30 min. The organic phase was separated, and the aqueous layer was extracted with EtOAc (3 × 30.0 mL). The combined organic layers were successively treated with NaHCO_3_ saturated aqueous solution, and NaCl saturated aqueous solution, and then anhydrified over Na_2_SO_4_, filtered and concentrated under reduced pressure. The crude product was purified by flash column chromatography by using Petroleum Ether/EtOAc (7:3) as eluent, followed by crystallization from EtOH. Orange powder, 73% yield, mp 109–111 °C. ^1^H NMR (400 MHz, DMSO-d_6_) 3.83 (s, 3H, OCH_3_), 6.12 (s, 1H, CH), 6.79 (d, 1H, *J* = 16.0 Hz, 1H, β-CH=C), 6.82 (d, *J* = 8.2 Hz, 1H, H-5), 6.95 (d, *J* = 16.0 Hz, 1H, β-CH=C), 7.16 (dd, *J* = 8.2, 2.0 Hz, 1H, H-6), 7.33 (d, *J* = 2.0 Hz, 1H, H-2), 7.57 (d, *J* = 15.9, Hz, 1H, α-CH=C), 7.60 (d, *J* = 15.9, Hz, 1H, α-CH=C), 7.64 (d, *J* = 8.6 Hz, 2H, 2′H and 5′H), 7.67 (d, *J* = 8.6 Hz, 2H, 3′H and 5′H). ^13^C NMR (101 MHz, DMSO-d_6_) δ 56.15, 102.01, 111.83, 116.15, 121.54, 123.83, 123.92, 125.55, 126.62, 130.54, 132.38, 134.54, 138.58, 142.17, 148.46, 150.07, 181.37, 185.63. ^1^H NMR (400 MHz, CDCl_3_) δ 3.96 (s, 3H, OCH_3_), 5.82 (s, 1H, keto-enol-CH), 5.85 (br s, 1H, OH), 6.50 (d, 1H, *J* = 16.0 Hz, CH=CH), 6.59 (d, 1H, *J* = 15.6 Hz, CH=CH), 6.94 (d, 1H, *J* = 8.0 Hz, Ar), 7.06 (d, 1H, *J* = 2.0 Hz, Ar), 7.13 (dd, 1H, *J* = 1.6 and 8.4 Hz, Ar), 7.41 (d, 2H, *J* = 8.0 Hz, Ar), 7.53 (d, 2H, *J* = 8.0 Hz, Ar), 7.54 (d, 1H, *J* = 16.0 Hz, CH=CH), 7.62 (d, 1H, *J* = 15.6 Hz, CH=CH). ^13^C NMR (101 MHz, CDCl_3_) δ 56.1, 102.5, 109.8, 115.0, 121.9, 124.3, 124.7, 127.8, 128.1, 129.8 (2C), 132.3 (2C), 134.0, 139.5, 140.0, 147.0, 148.0, 183.2 (2C). HRMS [+H^+^]: 401.0388 calculated for C_20_H_17_BrO_4_ (400.03). ESI-MS (*m*/*z*): 423 (M + Na)^+^, 425 (M + 2 Na)^++^.

### 4.4. Preparation of Aβ42 Oligomers

Aβ42 peptide solutions were prepared according to protocol #1 reported in [48]. Briefly, the Aβ42 peptide was solubilized in 1,1,1,3,3,3-hexafluoro-2-propanol (HFIP) and the solvent was evaporated via Eppendorf Concentrator plus^®^ (Haburg, Germany), after an appropriate incubation time. Peptide films were then redissolved in 20 mM NaH_2_PO_4_/Na_2_HPO_4_ pH 7.4 to obtain 221 μM Aβ42.

The resulting peptide solution was ultrafiltered (14,000× *g*, 60 min, 25 °C, as suggested by suppliers) on 50kDa-Amicon^®^ Ultra centrifugal filter units (Merck Millipore, Carrigtwohill, County Cork, Ireland). To approximately restore the original concentration, the recovery of the retained portion was achieved by reverse spinning (14,000× *g*, 5 min, 25 °C) by adding to the membrane a volume of 20 mM NaH_2_PO_4_/Na_2_HPO_4_ pH 7.4 equal to that of the ultrafiltrated sample.

Just before treating the cells with the isolated oligomeric fractions, total Aβ42, ultrafiltrated, and retained solutions were injected in CE, to verify the success of the ultrafiltration procedure and assign the oligomeric state. In the absence of standards, a proper quantitative evaluation of the oligomeric concentration from the electrophoretic peak area is intrinsically inaccessible. However, a semiquantitative comparison of the electropherograms obtained before and after ultrafiltration afforded an adequate control of satisfactory recovery. Cells were then treated with serial dilutions (1:10, 1:20, 1:40, 1:200 in culture medium) of filtrated (LMW Aβ42Os) and retained (LMW Aβ42Os) solutions. The molecular weight range for each species is clarified in the Discussion section.

### 4.5. Capillary Electrophoresis

Aβ42 peptide was solubilized by slightly modifying protocol #3 described in [48]. Briefly, lyophilized Aβ42 was dissolved in HFIP (1 mg/mL, 221 μM) and kept at 4 °C for 30 min. The solution was then aliquoted in microfuge tubes and the solvent was evaporated by Eppendorf Concentrator plus^®^. The Aβ42 aliquots were redissolved in a basic mixture (ACN/300 μM Na_2_CO_3_/113 mM NaOH, 48.3:48.3:3.4 *v*/*v*/*v*) to obtain 500 μM Aβ42. This solution was then diluted to the operative concentration (221 μM Aβ42 control peptide) with 20 mM NaH_2_PO4/Na_2_HPO4 pH 7.4, with or without small molecules. Stock solutions of curcumin and curcumin-based derivatives (1.53 mM) were prepared in pure ethanol.

For coincubation studies, a 500 μM Aβ42 solution in the basic mixture was diluted with a proper amount of phosphate buffer containing the compound, to obtain a compound concentration of 10 or 1 μM.

The monitoring of the Aβ42 aggregation process in the absence and presence of curcumin and curcumin analogues was accomplished by an Agilent Technologies 3D CE system with a built-in diode-array detector (Waldbronn, Germany). For the separation, a fused silica capillary (Polymicro Technologies, Phoenix, AZ, USA) (33 cm, 24.5 cm to the detector) was employed. The background electrolyte (BGE) consisted of 80 mM NaH_2_PO_4_/Na_2_HPO_4_ pH 7.4. Samples were injected by applying a pressure of 30 mbar for 3 s at the anodic end of the capillary. Separations were carried out at 12 kV (operative current: 80–85 μA) with the positive electrode at the inlet. The detection wavelength was set at 200 nm and the capillary temperature was kept constant at 25 °C. A perturbation of the baseline due to the sample solvent mixture was taken as a measure of the electroosmotic flow (EOF). Oligomeric species were identified based on their effective mobility (μeff), which was calculated by subtracting the contribution of the EOF from the apparent mobility (μapp). Semiquantitative analyses were performed based on the normalized area % [48].

### 4.6. Primary Cell Cultures

All experiments were performed in accordance with EU Directive (2010/63/EU) for the care and use of laboratory animals and those of the Italian Ministry of Health (D.L. 26/2014) and were approved by the Institutional Review Board for Animal Research of the University of Padua and by the Italian Ministry of Health (protocol number 41451.N.N8P).

For the primary microglia cell cultures, microglial cells were isolated from mixed glial cell cultures prepared from the cerebral cortices of postnatal day 1 Sprague-Dawley rat pups (CD strain), plated in 75 cm^2^ poly-L-lysine-coated tissue culture flasks (1.5 brains per flask), and grown in high-glucose Dulbecco’s modified Eagle’s medium (DMEM) with 2 mM glutamine, 10% heat-inactivated FBS, 100 units/mL penicillin, 100 μg/mL streptomycin, and 50 μg/mL gentamycin (growth medium) [74]. Seven days after isolation, mixed glial cultures reached confluence, and microglia were separated from the astroglial monolayer by shaking (200 rpm for 1 h at 37 °C), re-suspended in a growth medium, and plated on poly-L-lysine-coated plastic wells at a density of 1.50 × 10^5^ cells/cm^2^. Cells were allowed to adhere for 45 min and then washed to remove non-adhering cells. Cultures obtained using this procedure generated 97% microglia immunopositive to a primary polyclonal antibody against ionized calcium-binding adaptor molecule 1 (Iba1, 1:800, Wako Chemicals USA Inc., Richmond, VA, USA, Cat. 019-19741), a marker for microglia cell types (Supplementary Figure S5). Cells were maintained at 37 °C in a humidified atmosphere containing 5% CO_2_ and 95% air.

For the primary cortical neuronal cell cultures, primary cortical neurons were isolated from sacrificed Sprague Dawley pregnant female rats, and cortical cultures were prepared from E17-E18 embryos as described previously [75]. Briefly, after removal of meninges, cerebral cortices were dissected and dissociated with a papain tissue dissociation kit (Worthington, Lakewood, NJ, USA) following the manufacturer’s instructions. Dissociated cells were resuspended in a Neurobasal medium containing 2% B27 supplement, 1 mM sodium pyruvate, 2 mM L-glutamine, 100 units/mL penicillin, 100 μg/mL streptomycin, and 50 μg/mL gentamicin (plating medium), then plated on poly-D-lysine-coated 24-well plates at a density of 2 × 10^5^ cells/well in 1 mL. After two days, cultures received 1 mL/well of plating medium but containing B27 supplement without antioxidants. Cells were maintained at 37 °C in a humidified atmosphere containing 5% CO_2_ and 95% air and used for treatment on day seven. For indirect toxicity experiments, cells were treated for 24 h with a 10% primary microglia-conditioned medium that was mixed with a 90% plating medium.

### 4.7. Preparation of Microglia-Conditioned Media

Microglia were pretreated for 1 h with curcumin and curcumin analogues before stimulation with LMW or HMW Aβ42 for 24 h. Then, conditioned media were collected and centrifuged to remove cells and debris. Medium from vehicle-treated microglia was used as a control (CM-CTR). Microglia-conditioned medium was added to neurobasal medium to obtain a final ratio of 1:10.

### 4.8. Lactate Dehydrogenase (LDH) Assay

The direct and indirect toxicity of Aβ42 oligomers was measured as lactate dehydrogenase (LDH) release, using the CytoTox^®^ non-radioactive cytotoxicity assay kit (Promega Corporation, Madison, WI, USA) following the manufacturer’s instructions [76].

### 4.9. MTT Assay

Microglial cell viability was evaluated by a quantitative colorimetric method utilizing the metabolic dye 3-(4,5-dimethylthiazol-2-yl)-2,5-diphenyltetrazolium bromide (MTT) [25,77]. Briefly, cells were seeded in 96-well plates and treated with curcumin, **cur6**, and **cur16** (1–50 µM). After 24 h incubation, the medium was removed and the cells were incubated with MTT (0.18 mg/mL) in a humidified incubator at 37 °C for 4 h. Afterward, the supernatants were removed, and the formazan crystals developed in the viable cells were solubilized with DMSO. The plates were then read on a microplate reader (Victor2 Multilabel Counter, Wallac, Cambridge, MA, USA) using a test wavelength of 570 nm and a reference wavelength of 630 nm. Cell viability was expressed as the percentage of viable cells relative to that of control cells.

### 4.10. Cytokine Determination

After treatments, culture media were collected and IL-1β and TNF-α assayed using commercially available ELISA kits, according to the manufacturer’s instructions. Cytokine concentrations (pg/mL) in the medium were determined by reference to standard curves obtained with known amounts of IL-1β or TNF-α.

### 4.11. Morphological Analysis of Apoptosis by DAPI Staining

To monitor apoptosis, DAPI staining was performed. At the end of treatments, cells were fixed with 4% paraformaldehyde (pH 7.4, for 15 min at room temperature), permeabilized with 0.1% Triton X-100 in PBS for 1 h, and finally stained with 4′,6-diamidino-2-phenylindole (DAPI; 0.1 μg/mL) for 3 min in a dark condition. Coverslips were then mounted on microscope slides with Fluoromount-G mounting medium (Fisher Scientific, Milan, Italy). Fluorescent images were captured with a digital camera (DC 200) connected to a fluorescence microscope (DMR), both from Leica Imaging Systems (Cambridge, UK), and microscope settings were kept constant for all images to permit a direct comparison of all images.

### 4.12. Immunofluorescence

Cells were fixed with 4% paraformaldehyde (pH 7.4, for 15 min at room temperature) and subsequently, non-specific staining was blocked by incubating with 5% normal goat serum/0.1% Triton X-100 in PBS (blocking solution) for 1 h at room temperature. Cells were then incubated with the primary antibody anti-βIII tubulin (1:500) for 2 h at room temperature. Then, cells were extensively washed with PBS and incubated with the Alexa Fluor 488 secondary antibody (1:1000) for 1 h at room temperature. Both antibodies were diluted in the blocking solution. Immunostaining control included omission of the primary antibody. Coverslips were then mounted on microscope slides with Fluoromount-G mounting medium (Fisher Scientific, Milan, Italy). Fluorescent images were captured as described in Section 4.11.

### 4.13. Quantification of Neuritic Beading

Images were analyzed with ImageJ software (National Institutes of Health, Bethesda, MD, USA) using the “analyze particles” method. To each image, the “threshold” function was applied to convert the fluorescent images into binary black and white images. The positively labeled axons were identified using the Otsu threshold algorithm [78]. This method is an objectively applied algorithm that weighs the intensity of the background against the positive staining. After applying the threshold settings, the “analyze particle” function was used with the size (μm^2^) set from 0.5–1.50 and circularity set from 0.75–1.0 to include all spheroidal particles and exclude axon fragments with the same area. Quantification was obtained from five random fields for each condition, from three independent experiments.

### 4.14. Statistical Analysis

All data represent the results of at least three independent experiments. Data were analyzed using GraphPad Prism Software, version 6.0 (GraphPad Software, Inc., San Diego, CA, USA). Results are expressed as mean ± SEM. Statistical analyses to determine group differences were performed by one-way analysis of variance (ANOVA) followed by a post hoc Holm–Sidak’s test or by two-way ANOVA with Tukey’s post hoc test when appropriate. A value of *p* < 0.05 was considered to indicate statistically significant differences.

## Data Availability

The data presented in this study are available on request from the corresponding author.

## Supplementary Materials

The following supporting information can be downloaded at: https://www.mdpi.com/article/10.3390/ijms23084381/s1.
