# Supplementary material for: Modulation of Amyloid β-Induced Microglia Activation and Neuronal Cell Death by Curcumin and Analogues
Source: Int J Mol Sci. 2022 Apr 15;23(8):4381. doi: 10.3390/ijms23084381 (PMC9027876; doi:10.3390/ijms23084381)
Supplement: Supplementary file 1 [file ijms-23-04381-s001.zip › ijms-1650248-supplementary.pdf]

International Journal of *Molecular Sciences*

MDPI

Article

Modulation of Amyloid β-Induced Microglia Activation and Neuronal Cell Death by Curcumin and Analogues

Ersilia De Lorenzi [1], Davide Franceschini [2], Cecilia Contardi [1], Rita Maria Concetta Di Martino [3,†], Francesca Seghetti [3], Massimo Serra [1], Federica Bisceglia [1], Andrea Pagetta [2], Morena Zusso [2,*] and Federica Belluti [3]

[1] Department of Drug Sciences, University of Pavia, 27100 Pavia, Italy; ersidelo@unipv.it (E.D.L.); cecilia.contardi01@universitadipavia.it (C.C.); massimo.serra@unipv.it (M.S.); federica.bisceglia@unipv.it (F.B.)
[2] Department of Pharmaceutical and Pharmacological Sciences, University of Padua, 35131 Padua, Italy; dfrancesc@gmail.com (D.F.); andrea.pagetta@unipd.it (A.P.)
[3] Department of Pharmacy and Biotechnology, Alma Mater Studiorum-University of Bologna, 40126 Bologna, Italy; rita.dimartino@uniupo.it (R.M.C.D.M.); francesca.seghetti2@unibo.it (F.S.); federica.belluti@unibo.it (F.B.)
* Correspondence: morena.zusso@unipd.it; Tel.: +39-049-8275088
† Present address: Department of Pharmaceutical Sciences, University of Piemonte Orientale, Largo Donegani 2, 28100 Novara, Italy.

Citation: De Lorenzi, E.; Franceschini, D.; Contardi, C.; Di Martino, R.M.C.; Seghetti, F.; Serra, M.; Bisceglia, F.; Pagetta, A.; Zusso, M.; Belluti, F. Modulation of Amyloid β-Induced Microglia Activation and Neuronal Cell Death by Curcumin and Analogues. *Int. J. Mol. Sci.* **2022**, *23*, 4381. https://doi.org/10.3390/ijms23084381

Academic Editors: Marco Diociaiuti and Claudio Frank

Received: 8 March 2022
Accepted: 13 April 2022
Published: 15 April 2022

Publisher's Note: MDPI stays neutral with regard to jurisdictional claims in published maps and institutional affiliations.

Copyright: © 2022 by the authors. Licensee MDPI, Basel, Switzerland. This article is an open access article distributed under the terms and conditions of the Creative Commons Attribution (CC BY) license (https://creativecommons.org/licenses/by/4.0/).

Supplementary Materials

Table of Contents

Figure S1 2
Figure S2 2
Figure S3 3
Figure S4 3
Figure S5 4
Table S1 4

Materials and Methods 5
Physicochemical Property Prediction for Curcumin and **cur6** and **cur16** Analogues 5

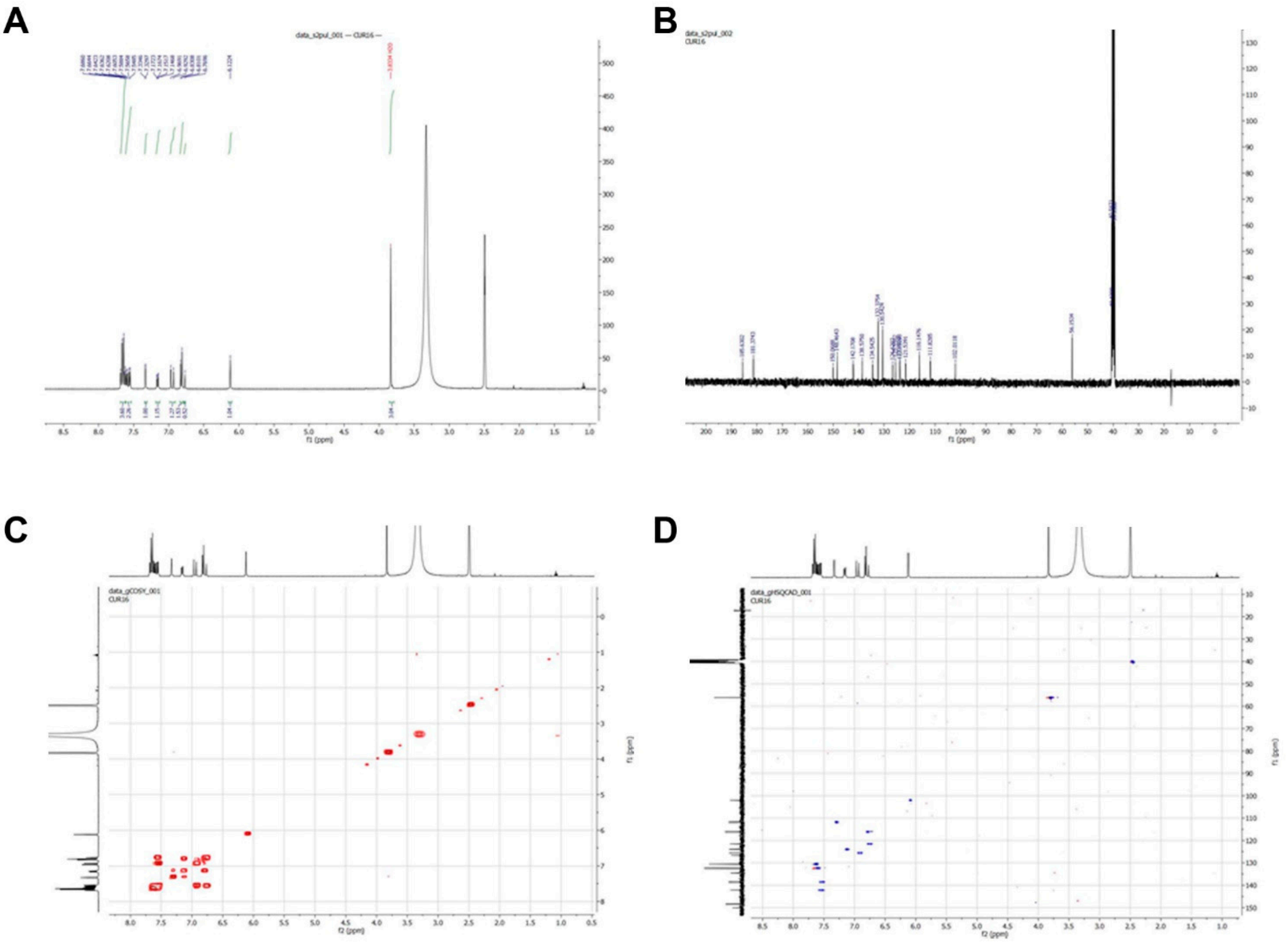

Figure S1. 1D and 2D NMR Spectra of **cur16**. (**A**) ^{1}H NMR (DMSO, 400 MHz) spectrum. (**B**) ^{13}C NMR (DMSO, 101 MHz) spectrum. (**C**) ^{1}H–^{1}H COSY spectrum. (**D**) ^{1}H-^{13}H HSQC spectrum.

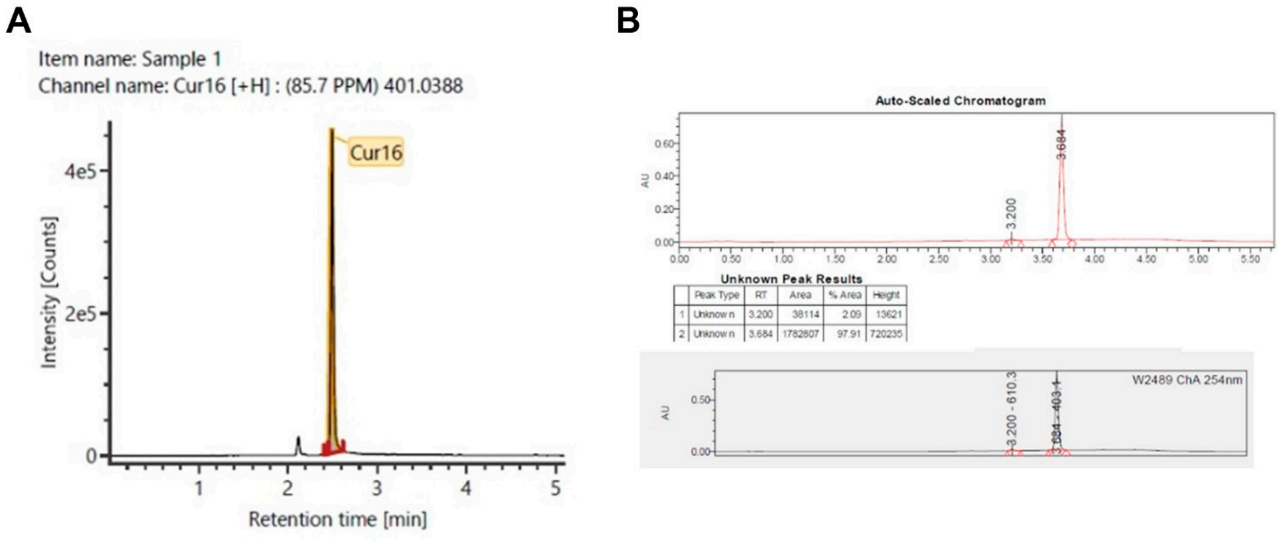

Figure S2. (**A**) HRMS spectrum. (**B)** UPLC-ms chromatogram.

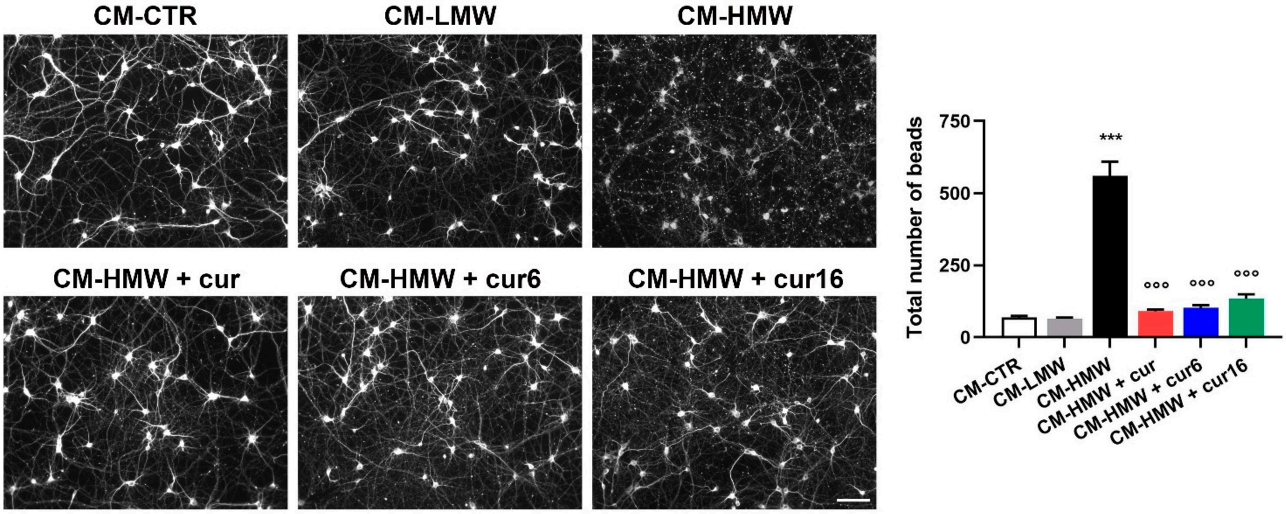

Figure S3. Effect of microglia-conditioned media on neuritic beading. Primary neurons were cultured from E17 rat embryos and after 7 days *in vitro* were exposed for 24 h to conditioned media from microglia untreated (CM-CTR), treated with 1:20 dilution of LMW (CM-LMW) or HMW (CM-HMW) Aβ42Os, or pre-treated with 10 μM curcumin (cur), **cur6**, or **cur16** before stimulation with HMW (1:20 dilution) Aβ42Os for 24 h. Cells were then processed for βIII-tubulin immunostaining. Experiments were performed 3 times and representative fluorescence microscopy images are shown. Scale bar is 50 μm. Quantification of neuritic beading was performed using ImageJ software as described in Materials and Methods, Section 4.12. Data are means ± SEM from five random fields of 3 independent experiments. ***$p < 0.001$ *versus* CM-CTR; °°°$p < 0.001$ *versus* CM-HMW. One-way ANOVA followed by Holm-Sidak's multiple comparison test.

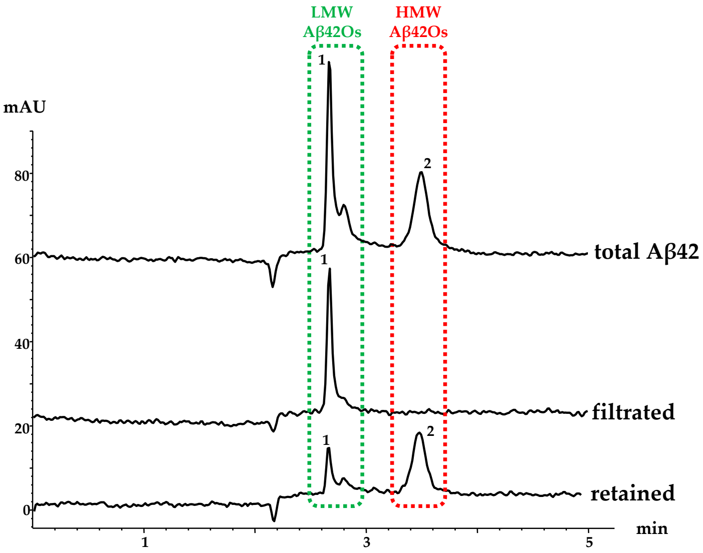

Figure S4. Capillary electrophoresis separation of Aβ42 oligomers. Representative electropherograms of: 221 μM total Aβ42 peptide analyzed by CE immediately after solubilization; Aβ42 filtrated fraction; Aβ42 retained fraction, recovered by reverse spinning. A 50 kDa-cutoff membrane was used. Experimental details are reported in Materials and Methods, Section 4.4.

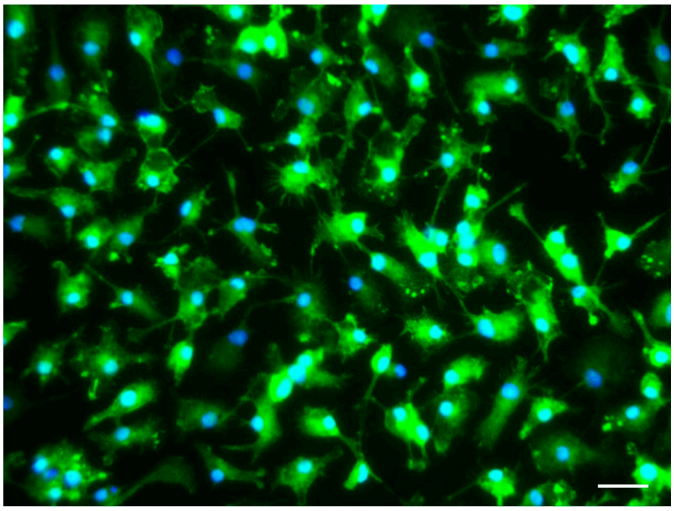

Figure S5. Primary microglial cell cultures. Cells were stained with anti-Iba1 antibody (green) and nuclei with DAPI (blue). Scale bar, 20 µm.

Table S1. Predicted physicochemical properties of **cur16**, **cur6**, and curcumin (FAF_Drugs4 tool).

ID	MW	logP	logD	logSw	tPSA	RotatableB	RigidB	Flexibility	HBD	HBA	HBD_HBA	Rings	MaxSizeRing
**cur16**	401,25	5,08	4,88	-5,32	66,76	6	16	0,27	2	4	6	2	6
**cur6**	406,47	5,83	5,35	-5,6	75,99	9	17	0,35	2	5	7	2	6
**curcumin**	368,38	3,98	3,65	-4,36	96,22	7	16	0,3	3	6	9	2	6

Materials and Methods

*Physicochemical Property Prediction for Curcumin and **cur6** and **cur16** Analogues*

The online server FAFDrugs4 (server available at http://fafdrugs4.mti.univ-paris-diderot.fr) was used to predict physicochemical properties of **cur6**, **cur16** and **curcumin** including logP, logD (at pH 7), flexibility, aqueous solubility (logSw), number of rotatable bonds, hydrogen-bond acceptors (HBAs), and hydrogen-bond donors (HBDs). In detail, this structural modification was performed taking advantage of FAF-Drugs4 tool helpful in predicting compounds' physicochemical properties. Thus, by comparing **cur6** and **cur16** predicted parameters (see Table S1) the performed modification led to a decrease in logP value (**cur6:** 5.83, **cur16:** 5.05), resulting in a presumed better solubility and a negligible impact on the number of putative H-bond donor and H-bond acceptor groups (**cur6**: 7, **cur16**: 6). Indeed, for **cur16** reduction in tPSA (**cur6**: 75.99, **cur16:** 66.76), rotatable bonds (**cur6:** 9, **cur16:** 6), and flexibility (**cur6**: 0.35, **cur16**: 0.27) were observed.
